# The Threat of Bis(2-Ethylhexyl) Phthalate in Coastal and Marine Environments: Ecotoxicological Assays Using Tropical Species from Different Trophic Levels

**DOI:** 10.3390/ijerph22030402

**Published:** 2025-03-10

**Authors:** Fernanda Silva dos Santos, Agatha Miralha, Amanda C. S. Coração, Antonio J. S. Rodrigues, Gabriel Kauai, Geovanna T. Borsato, Jéssica S. Costa, Julia de Morais Farias, Kettollen Brenda Ribeiro Pereira, Odilon Feuvrier, Rodrigo A. F. Silva, Nathália Rodrigues, Raquel A. F. Neves

**Affiliations:** 1 Research Group of Experimental and Applied Aquatic Ecology, Federal University of the State of Rio de Janeiro (UNIRIO), Rio de Janeiro 22290-240, Brazil; fesilvasantos@id.uff.br (F.S.d.S.); htamiralha@edu.unirio.br (A.M.); antoniorodrigues@edu.unirio.br (A.J.S.R.); nathalia.silva@edu.unirio.br (N.R.); 2 Graduate Program in Neotropical Biodiversity (PPGBIO), Institute of Biosciences (IBIO), Federal University of the State of Rio de Janeiro (UNIRIO), Rio de Janeiro 22290-240, Brazil; amandac.t@hotmail.com (A.C.S.C.); gabriel.kauaicamara@edu.unirio.br (G.K.); geovannatheobald@edu.unirio.br (G.T.B.); jscosta@edu.unirio.br (J.S.C.); julia.morais@edu.unirio.br (J.d.M.F.); kettollen@edu.unirio.br (K.B.R.P.); rodrigoaf.silva@edu.unirio.br (R.A.F.S.); 3 Département de Biologie, École Normale Supérieure de Lyon, 15 Parvis René Descartes, 69342 Lyon, France; odilon.feuvrier@ens-lyon.fr

**Keywords:** aquatic systems, biodegradation, ecotoxicology, lethality, phthalates, plasticizers, sublethality

## Abstract

Plastic and plasticizer pollution has been a concern worldwide over the past decade. Bis(2-ethylhexyl) phthalate (DEHP) is the most produced plasticizer and has been detected in coastal and marine ecosystems. This study aimed to assess the toxicity of acute exposure (24, 48, 72, and 96 h) to DEHP concentrations (0.045–6.00 mg·L^−1^) on marine and estuarine tropical species from distinct trophic levels. The lethality and sublethal responses were assessed on two microorganisms and three invertebrates, independently. The microorganisms—the microalga *Tetraselmis* sp. and the microbial consortium MP001—showed high tolerance and a density-rising tendency during exposure to DEHP. Among the invertebrates, the mortality of the brine shrimp *Artemia* sp. and the amphipod *Apohyale media* rose with increasing DEHP concentrations. However, *A. media* was more sensitive across time since its lethality reached 100% in almost all DEHP concentrations from 72 h. The dark false mussel *Mytilopsis leucophaeata* was the most tolerant invertebrate: no significant lethality (≤20%) was observed exclusively from 72 h of exposure to DEHP at intermediate–high concentrations. *Artemia* sp. and *M. leucophaeata* presented sublethal responses that seem to be good endpoints for ecotoxicological assays. These results reinforce evidence of DEHP contamination risks for tropical coastal ecosystems, as well as suggest candidate species for its biodegradation.

## 1. Introduction

Since the 1950s, plastic pollution has been increasing dramatically worldwide. The amount of plastic materials and their additives (e.g., plasticizers) has exceeded the capacity to manage them effectively in the environment [1,2,3]. Plastic and plasticizer input and their effects primarily on coastal and marine ecosystems have been concerns over the past decade [1,3]. Industrial and urban areas are probably the main sources of plasticizers in coastal and marine ecosystems [4]. Plastic production and other industries frequently represent point sources with direct or indirect release of plasticizers into the water bodies. Plasticizers can also be leached from plastic items employed in other anthropic activities, e.g., waste disposal sites and wastewater treatment plants [4,5]. Afterward, these chemicals can reach the rivers directly or via surface runoff and groundwater and eventually be transported to the sea. Moreover, due to their hydrophobic properties, plasticizers tend to adsorb onto plastic debris (i.e., macro- and microplastics), which further act as vectors transporting them over large distances in aquatic ecosystems [4,5].

The plastic additives most frequently found in marine compartments are bisphenol-A (BPA) and phthalates [6], of which phthalates represent around 92% of the world’s total additives [7]. Bis(2-ethylhexyl) phthalate (DEHP) is the most produced plasticizer which accounts for nearly 50% of the total amount of additives [7], reaching approximately 2 million tons annually. It provides flexibility and durability to plastic materials, which makes DEHP a commonly used compound in numerous industries, including cosmetics, clothing, toys, and PVC membranes [8,9]. The large-scale production of these items combined with their unmanaged disposal and the chemical properties of DEHP, make these contaminants ubiquitous in aquatic environments [10,11,12].

Several studies have detected DEHP in coastal and marine compartments, including the water column, sediments, and even organisms [13,14,15,16,17,18,19]. Concentrations of DEHP in such environments usually range from 0.002 µg·L^−1^ to 168 µg·L^−1^ in different locations, including the Mediterranean Sea, the Yellow Sea, the North Sea, the Pacific Ocean, and the Indian Ocean [13,14,15,16,17,18,19]. Little is known about DEHP concentrations along South America’s coastlines; however, phthalates (including DEHP) were recently detected in sediments from coastal and marine ecosystems of Rio de Janeiro city, Brazil [19].

Once in the aquatic environment, plasticizer properties drive its leaching, partitioning, and availability. The higher the molecular weight of phthalates is, the more lipophilic they are. Accordingly, lipophilic phthalates (e.g., DEHP) present lower leaching rates from the plastic pieces to the water column [4,5]. On the other hand, they may leach and bind rapidly into sediments. Dissolved or suspended organic matter in the water column also increases this plasticizer’s leaching rate [4,5]. Environmental conditions such as increased temperature and UV light incidence also intensify plasticizer leaching [4,5]. Henkel [20] found that high salinities decreased the DEHP leaching rate from PVC, whereas turbulence increased it. Accordingly, it was estimated that DEHP was leached faster from PVC microplastics in rivers (t_1/2_ > 49 years) than in the ocean (t_1/2_ > 398 years). Nevertheless, in both ecosystems, these microplastics are a long-term source of phthalates [21].

As expected, longer-chain-length phthalates (e.g., DEHP) tend to deposit and bind more strongly to sediments. Hence, they are less available for biodegradation, which is the primary pathway for phthalate degradation [4,5]. Higher salinity also increases phthalate sorption onto sediments or suspended organic particles due to greater particulate aggregation [4,5,21]. The biodegradation rate of phthalates depends on the microbial community metabolism as well. Environmental factors influence the persistence of plasticizers by affecting their biodegradation rate (e.g., temperature, pH, dissolved organic matter, oxygen content, turbulence). Once detached from sediment, plasticizers and their degradation residues can be absorbed by organisms and available to the trophic chain [4,5,21].

Phthalates have been found in coastal and marine organisms at different trophic levels, such as crustaceans, molluscs, and fish [22,23,24,25,26,27,28,29,30,31]. Because of their lipophilic properties, these chemicals tend to bind to fat and muscle tissues. According to Billings [4], higher-molecular-weight phthalates (e.g., DEHP) have been detected at higher concentrations in aquatic organism tissues than low-molecular-weight phthalates. DEHP is an endocrine disruptor in males acting as an androgen antagonist, including in human beings. Adverse effects of DEHP include endocrine system disruption, reproductive dysfunction, growth inhibition, and lethality [9,10,32,33,34,35].

According to European Union Regulation (EC) [36], DEHP was included in the candidate list of Substances of Very High Concern (Repr. 1B) due to its endocrine-disrupting properties for human health. In Brazil, the use of DEHP in food-contact plastics has been restricted since 2020, with a maximum limit of 1.5 mg kg^−1^ for any food product (Agência Nacional de Vigilância Sanitária, ANVISA). However, given the widespread presence of DEHP in coastal and marine environments, seafood consumption remains a major route of human exposure [6,9]. Therefore, studies on the effects of plasticizers’ on aquatic organisms are crucial to understanding the threat of such chemicals in these environments and, ultimately, human health. This study aimed to assess the toxicity of acute exposure to DEHP on marine and estuarine tropical species from distinct trophic levels: a primary producer microalga, a microbial consortium, a zooplanktonic grazer, a benthic omnivorous invertebrate, and a suspensivorous filter-feeder. For this, the lethality and sublethal responses of the five tested organisms were evaluated, independently, during the exposure (24, 48, 72, and 96 h) to increasing concentrations of DEHP (0.045–6.00 mg·L^−1^). In addition to evaluating the sensitivity of previously unstudied species to DEHP, the present study highlights its toxic effects at different trophic levels, providing evidence of the risks for tropical coastal ecosystems. Moreover, this study aligns with the United Nations’ Sustainable Development Goal 14 (Life Below Water) by advancing research in marine sciences to improve marine health, as well as environmental and human safety.

## 2. Materials and Methods

### 2.1. Organisms

Five groups of aquatic organisms were chosen to evaluate DEHP effects during acute exposure. A microalgal species and a bacterial consortium represented the microbial sphere—a primary producer and a decomposer, respectively. Moreover, three invertebrates comprised the following trophic roles: a filter-feeder zooplanktonic grazer, a benthic omnivore, and a benthic suspensivorous filter-feeder. Two of them are already recommended species for toxicity tests in coastal aquatic systems: the chlorophyte microalga *Tetraselmis* sp. [37] and the brine shrimps *Artemia* sp. [38]. Although amphipods and mussels are also suggested groups for ecotoxicological tests by OECD [38], *Apohyale media* and *Mytilopsis leucophaeata*, respectively, have never been studied for this purpose, even though microplastics have already been found in both species [39,40]. The bacterial consortium was chosen as a non-standard group since it is a candidate for xenobiotics’ bioremediation [41] and could respond to DEHP exposure.

In contrast to standard toxicology assays that use only model species, ecotoxicological studies must consider testing combined species differing in food chain position to obtain more robust information on DEHP toxicity in tropical coastal systems [42]. The benthic invertebrates (e.g., *Apohyale media* and *Mytilopsis leucophaeata*) are more susceptible to the negative impact of DEHP because its highest levels are generally observed in bottom water layers near accumulation zones of plastic debris from where DEHP is leached [43]. Amphipods are a crucial component of food webs since they are herbivores, detritivores, micropredators, and scavengers [44]. Moreover, they are the main food for predatory fish [45]. In addition to their ecological importance, the large number of species and high densities of amphipods in many estuarine and soft-bottom marine ecosystems make them good organisms for ecotoxicological assays [44]. In the case of bivalves, their feeding mechanism alters nutrient dynamics [46] and contributes to clarity and light penetration into the water column [47,48], which affects phytoplankton dynamics [49]. Furthermore, *Mytilopsis leucophaeata* are known to tolerate wide ranges of temperature and salinity [50] and have a high tolerance to contaminants, being able to bioaccumulate and translocate them through the trophic chain [51].

**Figure 1:**
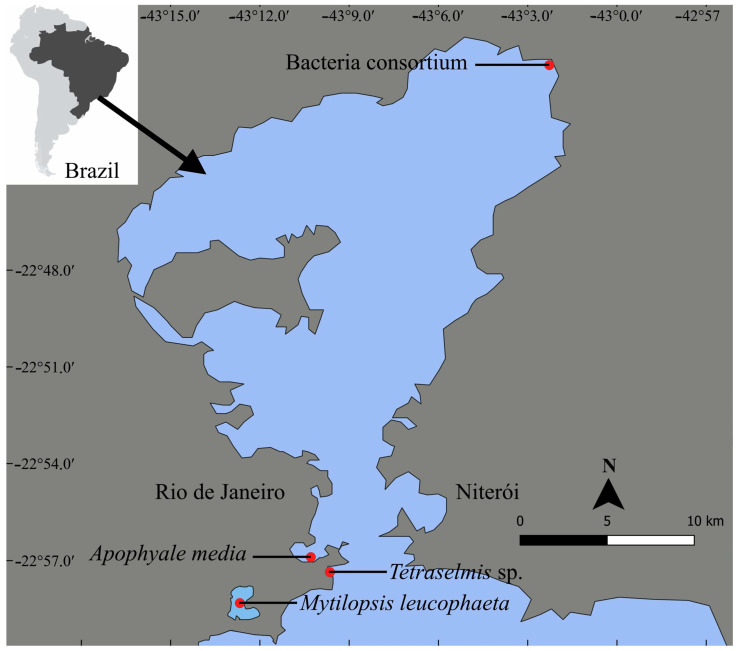
Geographical location of sampling sites of the microalga *Tetraselmis* sp., the bacterial consortium strain MP001, the amphipod *Apohyale media*, and the dark false mussel *Mytilopsis leucophaeata* at Guanabara Bay and Rodrigo de Freitas Lagoon in Rio de Janeiro state, southeast of Brazil. The brine shrimp *Artemia* sp. was purchased; thus, it was not shown here.

Sampling sites of each organism tested in this study are shown in Figure 1, except individuals of *Artemia* sp. that were obtained from a specialized store. The microalga *Tetraselmis* sp. (Chlorophyceae) was isolated by the single-cell method from Guanabara Bay, Rio de Janeiro, Brazil (22°46′05.73″ S, 43°10′04.31″ W). The clonal culture of *Tetraselmis* sp. was maintained at the Laboratory of Cultures and Experiments from the Federal University of the State of Rio de Janeiro (UNIRIO) in the following conditions: L1 medium [52] modified by omitting silicate, nickel, vanadium, and chromium, at 24 ± 2 °C and under 12:12 h (light:dark) photoperiod in temperature- and light-controlled Biochemical Oxygen Demand (B.O.D) Incubators (Solidsteel, BRA).

The bacterial consortium strain MP001 was isolated from the sediment of the Magé mangrove, Rio de Janeiro, Brazil (22°43′14″ S, 43°11′20″ W), which is localized in Guanabara Bay surroundings. The consortium was then enriched with petroleum (28°API crude-oil) for microorganism selection [41] and kept frozen in 50% glycerol (RPI, Troy, NY, USA) at the Multidimensional Water Microbiology Laboratory collection from the Federal University of the State of Rio de Janeiro (UNIRIO). MP001 consortium was activated in nutrient-rich TSB broth (casein peptone = 17.0 g, soy peptone = 3.0 g, D(+)-glucose = 2.5 g, NaCl = 5.0 g, and K_2_HPO_4_ = 2.5 g diluted in 1 L of distilled water; Kasvi, BRA [53]) at 37 °C and harvested by centrifugation (4300 rpm for 15 min at room temperature). Then, it was washed and re-suspended in 0.9% saline buffer (NaCl, Dinâmica, BRA).

The brine shrimps *Artemia* sp. were purchased from a local aquaculture store. Approximately 150 adult individuals were acclimatized for 48 h in 1 L aquaria filled with artificial filtered seawater (FSW; glass–fiber filter, Millipore AP-40, Millipore Brazil, São Paulo, Brazil), at salinity 35, 25 ± 1 °C, constant aeration, and a 12:12 h (light:dark) photoperiod. For the assay, they were sorted based on a standardized size (~7 mm).

The amphipod *A. media* was manually collected from macroalgae *Ulva* sp. on the right rocky shore at Vermelha Beach, Rio de Janeiro, Brazil (22°57′18.59″ S, 43°9′52.91″ W). Approximately 800 adult individuals were obtained and acclimatized in aerated artificial FSW (glass–fiber filter, Millipore AP-40, Millipore Brazil) at salinity 35, 25 ± 1 °C, and 12:12 h (light:dark) photoperiod in 1 L aquaria for 24 h. Individuals were selected based on a standardized size for the assay (~15 mm).

Clusters of the dark false mussel *M. leucophaeata* were collected by scraping the hard substrate at the surroundings of Rodrigo de Freitas Lagoon, Rio de Janeiro, Brazil (22°57′48.4″ S, 43°12′35.5″ W) and then transported to the laboratory facilities in a container (40 L) filled with the lagoon water. The shells of individuals ranging from 15 to 20 mm were cleaned with a soft sponge, and then they were acclimated in a 40 L aquarium (Boyu ZJ 401) containing aerated filtered brackish water (FBW; glass-fiber filter, Millipore AP-40, Millipore Brazil) at salinity 12.6, 25 ± 1 °C for 48 h.

During acclimation and independent assays, water salinity was kept constant similar to the value measured at the sampling site (thermosalinometer model HI98319) or kept at culture conditions for all tested organisms. For samplings, scientific research and collecting permits authorizing field studies were obtained from Chico Mendes Institute for Biodiversity Conservation (ICMBio, Brasília, Brazil), Brazilian Ministry of the Environment (permit number: 92128-1), and the National System for the Management of Genetic Heritage and Associated Traditional Knowledge (SISGEN n° A1B005D).

### 2.2. Stock and Test Solution Preparation

DEHP stock solution (40 mg·L^−1^) was prepared by diluting the commercial chemical solution of DEHP (Sigma-Aldrich, St. Louis, MO, USA, purity 98%; CAS number: 117-81-7; EC number: 204-211-0) in the appropriate volume of filtered ultrapure water in a volumetric flask. The stock solution was then stored in a methanol-decontaminated glass flask at room temperature in the dark. Exposure concentrations of DEHP were prepared by serial dilutions (i.e., reducing its concentration by a factor of two) of the stock solution using filtered seawater (FSW), filtered brackish water (FBW), or Mineral Salt Medium (MSM) according to the test organism. Exposure concentrations were chosen based on their environmental relevance [54,55,56] and results of previous toxicity tests using aquatic species (e.g., EC_50,48h_ *Daphnia magna*: 0.16 mg·L^−1^—ECOTOX Database, https://norecopa.no/3r-guide/ecotox-database/ (accessed on 24 July 2023); EC_50,72h_ microalga *Pseudokirchneriella subcapitata*: 0.003 mg·L^−1^ [38]).

### 2.3. Experimental Design

Acute toxicity of DEHP was assessed after 24, 48, 72, and 96 h of exposure, according to the Standard Evaluation Procedure guidelines [57,58]. Aquatic organisms were exposed to eight different concentrations of DEHP (0.045, 0.094, 0.187, 0.375, 0.750, 1.50, 3.00, and 6.00 mg·L^−1^). This contaminant is chemically stable at room temperature and its biodegradability in aerobic conditions is close to 29 days (~82%) (SDS according to EC n° 1907/2006 [36]). Thus, DEHP degradation and potential differences between nominal and detected concentrations were considered negligible in our short-term incubation (24–96 h). Negative controls consisted of the tested organisms incubated in artificial filtered seawater (AFSW) or artificial filtered brackish water (AFBW) without DEHP. For the assays, artificial seawater was prepared using a commercial powder mixture (Ocean Tech Premium Reef Salt) diluted in distilled water to reach salinity 35, then artificial seawater was filtered in a glass–fiber membrane using a vacuum system (AFSW; Millipore AP-40, Millipore Brazil). AFBW was obtained by adjusting the salinity of AFSW with filtered distilled water from 35 to 12.6. Then, AFSW and AFBW were stocked in two aquaria (40 L; Boyu ZJ 401, Boyu, Guangdong, China) with constant water circulation. All the toxicity tests were carried out using clean and sterilized glass laboratory supplies in a Biochemical Oxygen Demand (B.O.D) Incubator (Solidsteel, BRA) at 24 ± 2 °C, with a 12:12 h dark–light cycle and photon flux density of 60 μmol m^−2^s^−1^ provided by cool-white fluorescent tubes.

#### 2.3.1. Primary Producer Microalgae

A volume of 6.67 mL of the microalga *Tetraselmis* sp. suspension was incubated with 80 mL of the DEHP solutions or negative controls (AFSW) to reach the final concentration of 1.5 × 10^5^ cells·mL^−1^ in 250 mL Erlenmeyer glass flasks (three replicates per treatment). Homogenized 5 mL aliquots of each replicate were collected with an automatic pipette at the beginning of the incubation and after 24, 48, 72, and 96 h of exposure and then preserved with neutral Lugol iodine solution for cell counting. Cell density was obtained by counting *Tetraselmis* cells preserved in Lugol using a Neubauer counting chamber (4 analytical replicates) in a light-inverted microscope (Primovert, Zeiss, Munich, Germany). Cell density (cell·mL^−1^) was converted into growth rate (GR), following the equation:

GR (day^−1^) = (Ln N_1_ − Ln N_0_)/(T_1_ − T_0_)

in which N_1_ is the cell density at the time 1, N_0_ represents the cell density at the beginning of incubation, and T_1_ − T_0_ is the incubation time interval evaluated in days.

After 96 h of exposure, an aliquot of 50 mL of each replicate was filtered in a glass fiber membrane using a syringe attached to a swinex (Merck, AP-40, 0.7 µm). The membranes were kept identified and frozen (−20 °C; Consul, BRA) for further chlorophyll *a* analysis following the standard method according to CETESB [59]. Chlorophyll *a* was extracted by macerating the membranes with 10 mL of acetone solution (90%) and kept on dark glass tubes at 4 °C overnight (~20 h). The supernatant was collected from tubes with a glass Pasteur pipette and transferred to a cuvette (3 mL) for the determination of chlorophyll *a* (µg·L^−1^) using a Trilogy Laboratory fluorometer (Turner Designs) equipped with a PN 7200-040 module (LED 460 nm, excitation ≤ 500 nm, and emission ≥ 665 nm). A chlorophyll *a* standard from spinach (Sigma-Aldrich, São Paulo, Brazil; CAS number 479-61-8) was diluted in acetone 90% to achieve a concentration of 10 mg·L^−1^. Before analysis, the fluorometer was calibrated using five concentrations of chlorophyll *a* standard obtained through dilutions of work solution in acetone 90% and a blank (only acetone 90%) following equipment instructions.

#### 2.3.2. Bacterial Consortium

The experimental units consisted of 125 mL Erlenmeyer glass flasks with 14 mL of DEHP solutions in mineral salt medium (MSM; NaCl = 1.0 g, K_2_HPO_4_·3H_2_O = 1.0 g, NH_4_Cl = 0.5 g, and MgSO_4_·7H_2_O = 0.4 g diluted in 1 L of distilled water; Dinâmica, BRA [58]), in triplicate per concentration, and 1 mL of the bacterial suspension inoculum (2.7 × 10^9^ cells·mL^−1^). At this point, DEHP was the sole carbon and energy source for bacteria. The negative control consisted of 14 mL of MSM and 1 mL of the bacterial inoculum performed in triplicate in Erlenmeyer flasks. After 24, 48, 72, and 96 h of incubation at 35 °C ± 1, aliquots of 1 mL were collected from each flask to estimate the bacterial optical density using absorbance data (600 nm) obtained by a Trilogy Laboratory Fluorometer (Turner Designs). The estimated growth rate (GR) was obtained using the following equation:

GR (day^−1^) = (Ln N_1_ − Ln N_0_)/(T_1_ − T_0_)

in which N_1_ is the absorbance at the time 1, N_0_ represents the absorbance at the beginning of incubation, and T_1_ − T_0_ is the incubation time interval evaluated in days.

#### 2.3.3. Zooplanktonic Grazer

Adult individuals of the brine shrimp *Artemia* sp. (n = 10) were placed in decontaminated glass Petri dishes (90 mm) filled with 20 mL of AFSW (control) or DEHP solutions in FSW. Three independent replicates were arranged for control and each tested concentration. Brine shrimp lethality and sublethal effects were monitored after 24, 48, 72, and 96 h exposure using a stereomicroscope (Leica EZ4 HD, GER, Leica Microsystems, Wetzlar, Germany).

To assess the sublethal effects, three categories of alterations were considered: 0 = no effect; 1 = slow swimming or appendages beating without movement; 2 = slow swimming and spasms; 3 = spasms, erratic swimming, and desynchronized beating of appendages; 4 = presence of tumors and/or epithelial necrosis. Sublethal effects were identified, and categories were summed up for each treatment replicate.

#### 2.3.4. Benthic Omnivorous Crustacean

Five individuals of the amphipod *A. media* were placed in decontaminated glass Petri dishes (90 mm) with 20 mL of AFSW (control) or DEHP solutions in AFSW in triplicate per concentration. After 24, 48, 72, and 96 h exposure, lethality and sublethal effects were assessed using a stereomicroscope (Leica EZ4 HD, GER).

Three categories of sublethal effects related to movement alterations were assessed: 0 = no effect; 1 = reduced swimming distance; 2 = longer jump response period. Sublethal effects were summarized for all categories in each treatment replicate.

#### 2.3.5. Suspensivorous Filter-Feeder

Five dark false mussels *M. leucophaeata* were arranged in 1 L aquaria filled with 300 mL of AFBW (control) or DEHP solutions in FBW (triplicate per concentration). Individuals of *M. leucophaeata* were monitored for lethal and sublethal responses after 24, 48, 72, and 96 h of exposure.

Lethality was recorded when the individuals were fully opened or floating in the water column, while sublethal effects were categorized based on three aspects, as follows: (a) response to stimulus through the assessment of valve closure reaction during needle contact: 0 = individuals completely closed, 1 = more than one second to respond to the stimulus, 2 = response to stimulus observed in less than one second; (b) byssus production: 0 = individuals that did not produce byssus, 1 = individuals that produced byssus either at the bottom of the aquarium or on other individuals; (c) cluster formation (i.e., aggregation behavior): 0 = individuals that did not form clusters, 1 = individuals that formed clusters. Sublethal effects were summed up for all categories in each treatment replicate.

### 2.4. Statistical Analysis

For each independent replicate by DEHP concentration and controls, growth rate and survival proportion were calculated by dividing the number of alive cells/individuals by the initial number in the prior day—t_0_, t_1_, t_2_, and t_3_. Then, the obtained values were used to calculate the arithmetic means to estimate the growth rate percentage (for microalga and bacterial consortium) or cumulative mortality (for invertebrate species).

A two-way Permutational Multivariate Analysis of Variance (PERMANOVA) was conducted to evaluate the influence of DEHP concentrations and exposure times (i.e., 24, 48, 72, and 96 h) microalga density (cells·mL^−1^) and estimated biomass of bacterial consortium (absorbance data) or mortality proportion of brine shrimps, amphipods, and mussels. Sublethal responses of invertebrates among treatments and exposure times were also assessed using two-way PERMANOVA analyses. Differences in chlorophyll concentration in microalga among DEHP treatments at the end of 96 h incubations were evaluated using one-way PERMANOVA. The PERMANOVA tests were based on Euclidean distances and 9999 permutations. Once the analysis reached the significance of *p* < 0.05, a pairwise *posteriori* comparison was applied. Statistical analyses and graphics were performed using the software PERMANOVA 1.6 (Anderson, 2005) and GraphPad Prism 8.02 (Graph Pad, San Diego, CA, USA), respectively.

## 3. Results

### 3.1. Primary Producer Microalgae

**Figure 2:**
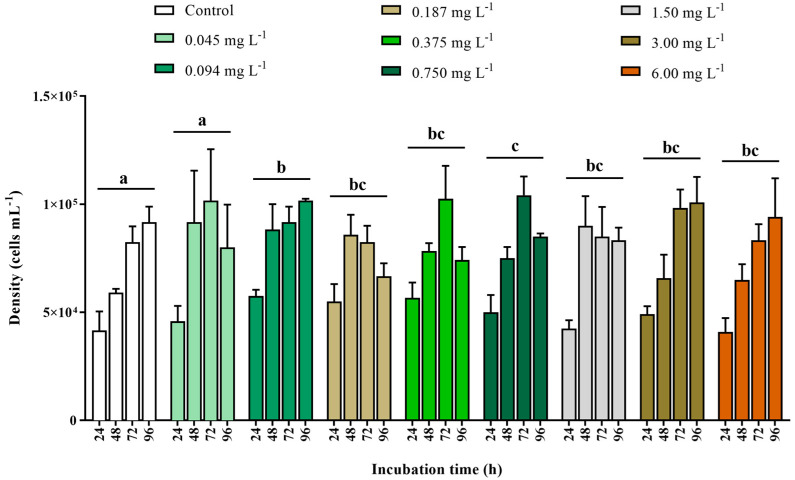
Density (cells·mL^−1^) of the microalga *Tetraselmis* sp. after 24, 48, 72, and 96 h incubation in control and DEHP treatments (0.045, 0.094, 0.187, 0.375, 0.750, 1.50, 3.00, and 6.00 mg·L^−1^). Data are shown as means ± standard error (n = 3 replicates per treatment and time). Distinct letters indicate significant treatment differences (pairwise comparison, *p* ≤ 0.043).

The density of the microalga *Tetraselmis* sp. tended to increase throughout the incubations, independently of DEHP concentration (Figure 2). However, no significant effect of incubation time was detected (PERMANOVA, F_3,107_ = 2.5353, *p* = 0.0644), nor the interaction between DEHP concentration and time (PERMANOVA, F_24,107_ = 1.360, *p* = 0.1604). A significant effect of DEHP concentration was found in *Tetraselmis* sp. density (PERMANOVA, F_8,107_ = 10.281, *p* = 0.0001) (Figure 2). The microalgal densities at the control and the concentration of 0.045 mg DEHP·L^−1^ were significantly lower than densities reached at all the other DEHP concentrations (pairwise comparisons, *p* ≤ 0.043). Still, no significant difference was found between the control and the lowest DEHP concentration (pairwise comparison, *p* = 0.927). These densities were statistically similar but different from all the others, so they were tagged with the same letter “a” for viewing purposes in Figure 2. The density of microalga exposed to 0.094 mg DEHP·L^−1^ was significantly higher than microalgal density at the concentration of 0.750 mg DEHP·L^−1^ (pairwise comparison, *p* = 0.0242). As the microalgal densities at these two concentrations were significantly different between them and, at the same time, significantly higher than that at control and the concentration of 0.045 mg DEHP·L^−1^, each was tagged with a different letter (b and c) in Figure 2. The densities at concentrations of 0.187, 0.375, 1.50, 3.00, and 6.00 mg DEHP·L^−1^ (tagged as “bc” in Figure 2) were statistically higher than that at control and the concentration of 0.045 mg·L^−1^ but did not significantly differ from those at concentrations of 0.094 and 0.750 mg DEHP·L^−1^. The average density reached by *Tetraselmis* sp. exposed to the 0.094 mg DEHP·L^−1^ was the highest in the assays (8.48 × 10^4^ cells·mL^−1^), while the lowest mean density was observed at the negative control (6.88 × 10^4^ cells·mL^−1^).

**Figure 3:**
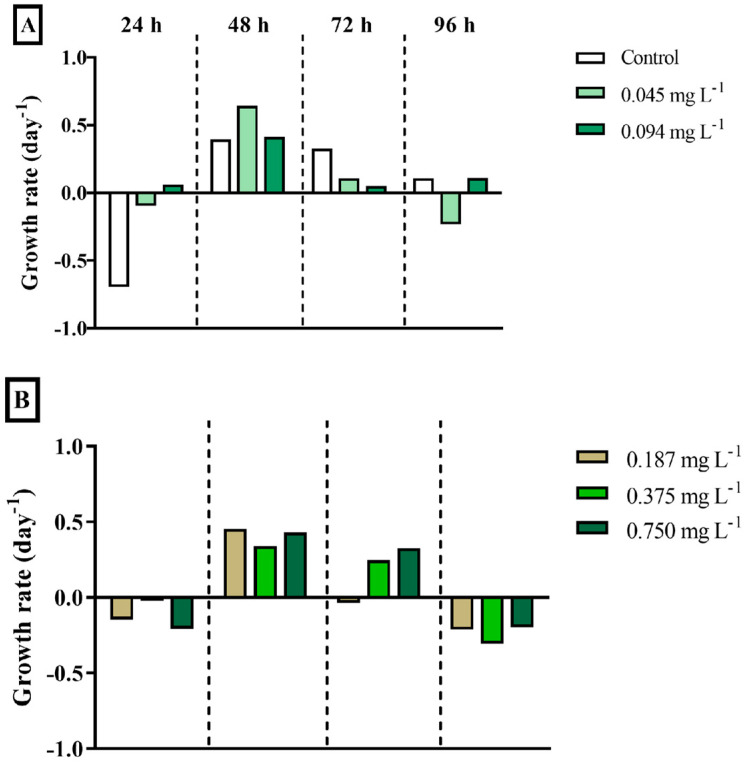

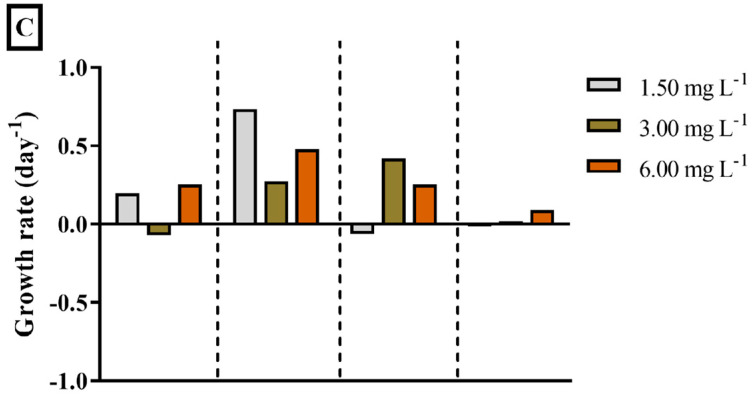
The growth rate (day^−1^) of the chlorophyte microalga *Tetraselmis* sp. exposed to different concentrations of DEHP after 24, 48, 72, and 96 h. (**A**) Data acquired at the control (only filtered artificial seawater), 0.045, and 0.094 mg·L^−1^; (**B**) Data acquired at the 0.187, 0.375, and 0.750 mg·L^−1^; (**C**) Data acquired at the 1.50, 3.00, and 6.00 mg·L^−1^. Data are presented as mean (n = 3 replicates per treatment and time). Negative values mean algal growth inhibition.

The exposure of the microalga *Tetraselmis* sp. to different concentrations of DEHP (0.045, 0.094, 0.187, 0.375, 0.750, 1.50, 3.00, and 6.00 mg·L^−1^) during four incubation times (24, 48, 72, and 96 h) did not inhibit its growth compared to negative controls (Figure 3). On the contrary, an increasing trend was observed, primarily after 24 h and 48 h of exposure. Therefore, growth rates presented the same pattern within the different exposure times, regardless of the concentration. That is, the growth rate of all concentrations fell after 24 h of exposure, increased after 48 h, and gradually fell again from 72 h to 96 h.

Finally, DEHP exposure did not affect the chlorophyll *a* (Chl*a*) concentration in the cells of *Tetraselmis* sp. after 96 h of exposure (PERMANOVA, F_8,26_ = 2.317, *p* < 0.0652). The mean Chl*a* concentration microalga in negative control was 292.79 µg·L^−1^, while in DEHP treatments, Chl*a* ranged from 278.88 at the higher DEHP concentration (6.00 mg·L^−1^) to 361.88 µg·L^−1^ at the treatment with 0.187 mg DEHP·L^−1^.

### 3.2. Bacterial Consortium

**Figure 4:**
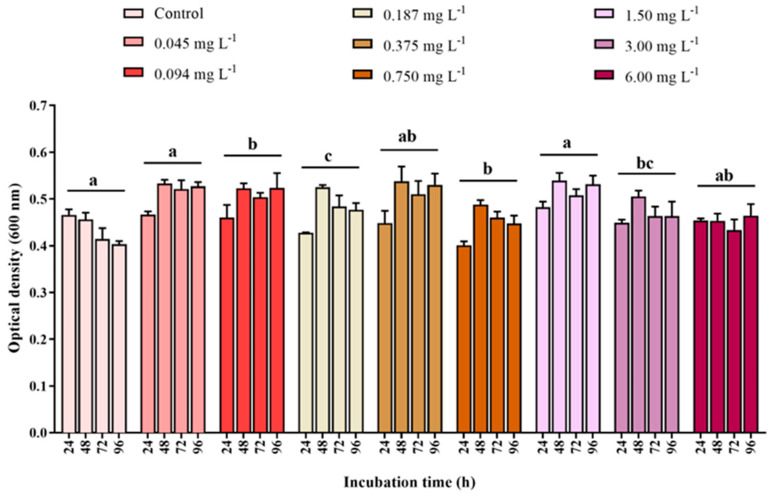
Optical density (absorbance at 600 nm) of the bacterial consortium strain MP001 after 24, 48, 72, and 96 h incubation in control and DEHP treatments (0.045, 0.094, 0.187, 0.375, 0.750, 1.50, 3.00, and 6.00 mg·L^−1^). Data are shown as means ± standard error (n = 3 replicates per treatment and time). Distinct letters indicate significant treatment differences (pairwise comparison, *p* ≤ 0.05).

Bacteria optical density showed a significant increase in DEHP treatments throughout the incubations, in contrast to the control (Figure 4). Significant effects of DEHP concentration (PERMANOVA, F_8,107_ = 9.1498, *p* = 0.0001) and the interaction between DEHP concentration and time were found for bacteria optical density (PERMANOVA, F_24,107_ = 4.0589, *p* = 0.0001; Supplementary Data). An isolated time effect was not detected on bacteria optical density (PERMANOVA, F_3,107_ = 0.8797, *p* = 0.4545). No significant difference was found among the optical densities of the consortium MP001 exposed to control and the concentrations of 0.045 and 1.50 mg DEHP·L^−1^ (pairwise comparison, *p* ≥ 0.4748). On average, these optical densities were lower compared with those at the concentrations of 0.094, 0.187, 0.750, and 3.00 mg·L^−1^ (pairwise comparisons, *p* ≤ 0.0383; Figure 4). As the densities at control and concentrations of 0.045 and 1.50 mg DEHP·L^−1^ were statistically similar, they are tagged with the same letter “a” for viewing purposes in Figure 4. The pattern of the optical densities at concentrations of 0.094 and 0.750 mg DEHP·L^−1^ was statistically similar (pairwise comparisons, *p* = 0.9009; labeled as “b” in Figure 4) but differed from that at 0.187 mg DEHP·L^−1^ (pairwise comparisons, *p* ≤ 0.056 considered; labeled as “c” in Figure 4). The concentrations of 0.375 and 6.00 mg DEHP·L^−1^ (tagged as “ab”) were not significantly different from those labeled as “a” or “b” (pairwise comparisons, *p* ≥ 0.0878), which include the control. Finally, the consortium density at concentration 3.00 mg DEHP·L^−1^ (tagged as “bc”) did not significantly differ from those labeled as “b” or “c” (pairwise comparisons, *p* ≥ 0.1203).

**Figure 5:**
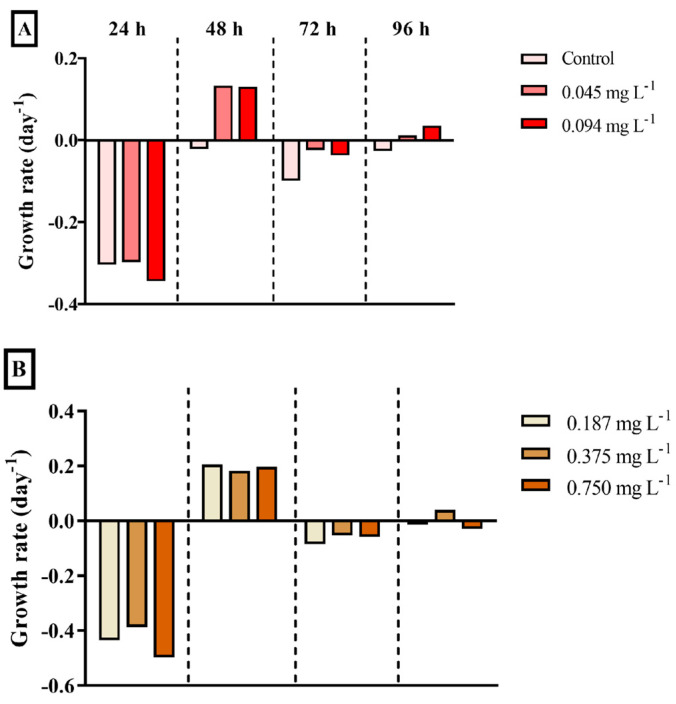

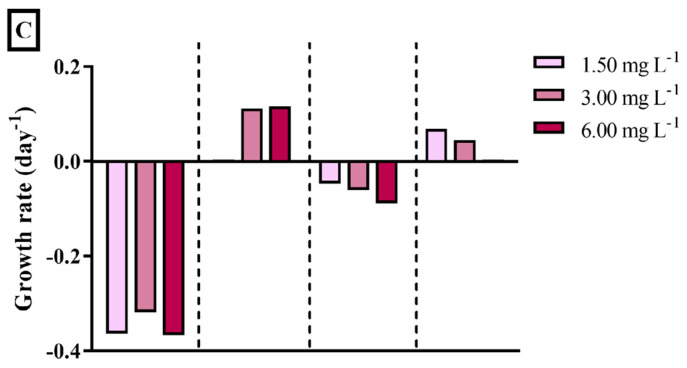
The estimated growth rate (day^−1^) of the bacterial consortium strain MP001 was exposed to different concentrations of DEHP during 24, 48, 72, and 96 h. (**A**) Data acquired at the control (only filtered mineral salt medium), 0.045, and 0.094 mg·L^−1^; (**B**) Data acquired at the 0.187, 0.375, and 0.750 mg·L^−1^; (**C**) Data acquired at the 1.50, 3.00, and 6.00 mg·L^−1^. Data are presented as a means (n = 3 replicates per treatment and time). Negative values mean bacterial growth inhibition.

The growth rate of bacteria was negative in control during all the exposure times, which indicates growth inhibition throughout the incubations (Figure 5). After 24 h and 72 h, the bacterial consortium presented a growth decline in all DEHP concentrations. However, a growth increase tendency was observed mainly after 48 h and, softly, after 96 h (Figure 5).

### 3.3. Zooplanktonic Grazer

The survival of the brine shrimp *Artemia* sp. was significantly affected by DEHP concentrations (PERMANOVA, F_8,107_ = 15.6002, *p* = 0.0001). However, there was no significant effect of the exposure time (PERMANOVA, F_3,107_ = 0.6743, *p* = 0.5681) and the interaction between concentration and time on this endpoint (PERMANOVA, F_24,107_ = 1.0751, *p* = 0.3971). Table 1 shows the cumulative percentage of mortality of *Artemia* sp. individuals after exposure to increasing DEHP concentrations. The survival of brine shrimps at the control significantly differed from those exposed to all DEHP concentrations (pairwise comparisons, *p* ≤ 0.0032), except at 0.045 and 0.094 mg·L^−1^. These two concentrations, in turn, presented statistically similar survival compared to the third and fourth concentrations (0.187 and 0.375 mg·L^−1^) and higher percentages than concentrations of 0.750, 1.50, 3.00, and 6.00 mg·L^−1^ (pairwise comparisons, *p* ≤ 0.0140). The brine shrimps exposed to intermediate concentrations (0.187, 0.375, 0.750 mg·L^−1^) and to 3.00 mg·L^−1^ of DEHP presented statistically similar survival. Finally, the survival rates in the concentrations of 1.50 and 6.00 mg·L^−1^ were similar and significantly lower than those of the others (pairwise comparisons, *p* ≤ 0.0060).

Regarding sublethal effects, the more frequently observed alterations were slow swimming and spasms, reported in almost all replicates from 0.094 mg·L^−1^ of DEHP concentration. Although epithelial necrosis was a symptom rarely seen among individuals, it was detected in brine shrimps exposed to 24 h (0.75 and 1.5 mg DEHP·L^−1^), 48 h (0.187, 0.375, 0.75 and 1.5 mg DEHP·L^−1^), 72 h, and 96 h (0.094, 0.187, 0.375, 0.750 and 1.5 mg DEHP·L^−1^). Epithelial necrosis was intense when present, and affected individuals showed swelling of the appendages, which interfered in their locomotion. DEHP exposure induced a significant effect on sublethal responses of *Artemia* individuals (Table 2; PERMANOVA, F_8,107_ = 3.77, *p* = 0.0009). Sublethal responses of brine shrimps exposed to the concentrations of 0.187, 0.750, and 6.00 mg DEHP·L^−1^ significantly differed from the control (pairwise comparisons, *p* ≤ 0.006). In addition, responses detected on brine shrimps exposed to 0.187 and 6.00 mg DEHP·L^−1^ also significantly differed from individuals exposed to the concentrations of 0.045, 0.094, and 3.00 mg DEHP·L^−1^ (pairwise comparisons, *p* ≤ 0.019). However, there was no significant time effect (PERMANOVA, F_3,107_ = 0.7549, *p* = 0.5195), and the interaction between time and concentration was detected on the sublethal responses of brine shrimps (PERMANOVA, F_24,107_ = 1.5607, *p* = 0.0773).

### 3.4. Benthic Omnivorous Crustaceans

The survival of the benthic amphipod *Apohyale media* was affected by DEHP concentrations (PERMANOVA, F_8,107_ = 12.1781, *p* = 0.0001) and by the interaction between concentration and time (PERMANOVA, F_24,107_ = 4.1953, *p* = 0.0001; Supplementary Data). Exposure time did not significantly affect the isolated amphipod survival (PERMANOVA, F_3,107_ = 2.0373, *p* = 0.1180). The survival of amphipods at the control was significantly higher compared to the individuals exposed to all the DEHP concentrations, except for the lowest DEHP concentration (0.045 mg·L^−1^; pairwise comparison, *p* = 0.5701). After 24 h, there was no mortality in the control, and the individuals at the lowest concentrations could hatch their eggs. In contrast, the amphipods exposed to the two highest concentrations of DEHP (3.00 and 6.00 mg·L^−1^, respectively) did not survive during this exposure time (Table 3). After 48 h of exposure, the mortality of individuals was higher compared to the first 24 h of incubation, even in the control. The survival analyses after 72 and 96 h of incubation revealed no live amphipods in almost all DEHP concentrations. No behavioral or morphological changes were detected in the amphipods’ *A. media* throughout incubations.

### 3.5. Suspensivorous Filter-Feeder

The dark false mussel *M. leucophaeata* survived at all treatments during the first 48 h. The lethality of *M. leucophaeata* individuals was only observed after 72 h of exposure to DEHP concentrations, reaching the highest cumulative mortality (20%) at the concentration of 1.50 mg DEHP·L^−1^ after 96 h (Table 4). A mortality of 7% was reported from 72 h exposure at the concentrations of 0.094 and 0.7502 mg·L^−1^, whereas mussels exposed to 0.187 and 3.00 mg·L^−1^ of DEHP presented the same percentage from 96 h. Despite the lethality observed in *M. leucophaeata* mussels during longer exposure periods (72 and 96 h), no significant effect of concentrations (PERMANOVA, F_8,107_ = 1.1561, *p* = 0.1592) and exposure time (PERMANOVA, F_3,107_ = 0.7698, *p* = 0.8496) was detected in mussel survival.

Concerning sublethal effects, the response of dark false mussels to the stimulus (Table 5) was significantly affected by treatment (PERMANOVA, F_8,107_ = 4.9472, *p* = 0.0001). No significant effect of time (PERMANOVA, F_3,107_ = 1.5706, *p* = 0.2000) and the interaction between treatment and time (PERMANOVA, F_24,107_ = 0.4350, *p* = 0.9887) was detected in the response of the mussels to the stimulus. The control differed from all the DEHP concentrations (pairwise comparison, *p* ≤ 0.0457), except the lowest DEHP concentration (0.045 mg·L^−1^; pairwise comparison, *p* = 0.2332). The lowest concentration, in turn, significantly differed from the higher ones (0.750, 1.50, 3.00, and 6.00 mg·L^−1^; pairwise comparison, *p* ≤ 0.05). The response of individuals to stimulus at the concentration of 0.187 mg DEHP·L^−1^ significantly differed from 0.750, 1.50, 3.00, and 6.00 mg DEHP·L^−1^ (pairwise comparison, *p* ≤ 0.05). A tendency of decrease in the indices (i.e., a delay in response time to the stimulus or valve closure) was observed at higher DEHP concentrations after 72 h (Table 5).

For byssus production (Table 5), significant differences were detected among treatments (PERMANOVA, F_8,107_ = 19.7191, *p* = 0.0001), exposure times (PERMANOVA, F_3,107_ = 3.9966, *p* = 0.0105), and the interaction between these two factors (PERMANOVA, F_24,107_ = 2.3960, *p* = 0.0023; Supplementary Data). The byssus production by individuals at the control and the lowest DEHP concentration (0.045 mg·L^−1^) differed from individuals exposed to the higher DEHP concentrations (0.750, 1.50, 3.00, and 6.00 mg·L^−1^; pairwise comparison, *p* ≤ 0.001). The byssus production by dark false mussels exposed to the DEHP concentrations of 0.094 and 0.187 mg·L^−1^ significantly differed from individuals exposed to 0.375, 0.750, 1.50, 3.00, and 6.00 mg DEHP·L^−1^ (pairwise comparison, *p* ≤ 0.05). In addition, the byssus production by individuals exposed to 0.375 mg DEHP·L^−1^ significantly differed from individuals at treatments of 0.750, 1.50, 3.00, and 6.00 mg DEHP·L^−1^ (pairwise comparison, *p* ≤ 0.0430). After 24 h and 48 h, there was a tendency to decrease byssus production by dark false mussels at all concentrations compared to controls. After 72 and 96 h, byssus production fell even in the control.

For cluster formation (Table 5), a significant effect was found for treatment (PERMANOVA, F_8,107_ = 6.46, *p* = 0.0001), exposure time (PERMANOVA, F_3,107_ = 2.77, *p* = 0.04), and the interaction between the treatment and exposure time (PERMANOVA, F_24,107_ = 1.6659, *p* = 0.0522). After 24 and 48 h, cluster formation by mussels fell abruptly at the lower DEHP concentrations (0.045, 0.094, 0.187 mg·L^−1^) than in the others and the control. After 72 and 96 h, cluster formation fell, even in the control.

## 4. Discussion

### 4.1. Primary Producer Microalgae

Microalgae growth is a widely used indicator of xenobiotic toxicity, and the chlorophyte *Tetraselmis* is a standard microalga for this purpose [38]. Although this genus has not been previously exposed to DEHP or other phthalates, it was tested against the plasticizer BPA [60,61,62,63,64]. Nevertheless, all these studies have found an inhibitory effect of BPA on *Tetraselmis* growth. Herein, *Tetraselmis* sp. showed high tolerance and a density-rising tendency during acute exposure (24–96 h) to increasing DEHP concentrations (0.045–6.00 mg·L^−1^). Moreover, the microalgal densities at the control and the concentration of 0.045 mg DEHP·L^−1^ were significantly similar and lower than those at all other tested concentrations. This growth tendency throughout increasing DEHP concentrations suggests a stimulatory effect, although the mechanism of which was not evaluated here. However, we hypothesize that the compound is taken up and then accumulated or metabolized by the chlorophyte cells. Hence, *Tetraselmis* sp. would degrade and use DEHP for growth and production, as it has been reported for microalgae exposed to other xenobiotics [65,66,67,68].

In this study, the growth rate of *Tetraselmis* sp. through exposure time was similar to a microalgae growth curve in a nutritional medium, regardless of the tested concentration (including control; [65]). That is, in the first 24 h of incubation, microalgae seemed to be at an adaptative phase with intense metabolic activity preparing for intense duplication (log phase) observed after 48 h. After 72–96 h, the growth rate fell gradually as the stimulation factor or nutrient source was consumed, corroborating our hypothesis. Nevertheless, the lower doses of DEHP seem to have greater stimulation over *Tetraselmis* sp., since the higher mean density (8.48 × 10^4^ cells·mL^−1^) was observed at incubation with 0.094 mg DEHP·L^−1^, followed by 7.98 × 10^4^ cells·mL^−1^ at 0.045 mg DEHP·L^−1^. It is worth noting that these concentrations have already been reported in the environment (≤0.168 mg·L^−1^) [12,13,14,15,16,17,18].

Chlorophyll *a* (Chl*a*) concentration reflects microalgae photosynthetic capacity and the physiological state of cells [69,70]. In agreement with the growth results, DEHP did not affect Chl*a* concentration in *Tetraselmis* sp. after 96 h of exposure, regardless of the concentration. Additionally, a higher mean Chl*a* concentration (361.88 µg·L^−1^) was reported at a concentration of 0.187 mg DEHP·L^−1^, suggesting intense cell activity at lower phtalate concentrations. Cell growth and Chl*a* concentration were evaluated for other microalgae after exposure to DEHP, namely the marine diatom *Chaetoceros decipiens-lorenzianus* [71], the dinoflagellate *Alexandrium pacificum* [72], and chlorophyta *Chlorella vulgaris* [73]. The cell density of *C. decipiens-lorenzianus* showed a slight, but significant (*p* < 0.05), increase after DEHP exposure, regardless of its concentration [68]. Concentrations of 1 and 10 μg DEHP·L^−1^ increased the growth rate of *C. decipiens-lorenzianus* to 1.11 a 0.98 day^−1^, respectively, compared to the experimental control (0.82 day^−1^; [70]). The dinoflagellate *A. pacificum* responded oppositely to the same DEHP concentrations tested (1 and 10 μg·L^−1^). *Alexandrium pacificum* was highly affected by the phthalate, with a decrease in cell density and Chl*a* concentration [72]. This inhibitory effect was also observed in the cell density of *C. vulgaris* exposed to the concentrations from 2 to 10 mg DEHP·L^−1^ [73]. Different responses of microalgae species to DEHP may alter natural phytoplankton communities, as previously observed [74]. Furthermore, it can also affect the dynamics of bloom algal events, including harmful algal blooms. Thus, depending on the bloom-forming species, DEHP’s presence can stimulate or inhibit them. Therefore, further studies testing the DEHP effect on other microalgae species are desirable.

The present results of cell growth and Chl*a* concentration obtained for *Tetraselmis* sp. exposed to DEHP highlight its high tolerance to this phthalate. Therefore, considering the stimulatory responses, this chlorophyta might be considered to be tested in further studies focusing on removal and biodegradation analyses as a potential species for bioremediation of marine or brackish aquatic environments contaminated with relevant DEHP concentrations that could promote a reduction of its availability in the environment [75,76]. Otto et al. [77] suggested that green microalgae could produce the enzyme laccase, which contributes to the breakdown of phenolic pollutants. Gattullo et al. [65] reported a good removal efficiency of BPA by the freshwater green alga *Monoraphidium braunii*. After the fourth day growth, *M. braunii* was able to remove 39, 48, and 35% of the BPA initial concentrations of 2, 4, and 10 mg·L^−1^, respectively. Yang [62] demonstrated that *Tetraselmis* sp. did not biodegrade compounds such as BPA and nonylphenol (NP); however, this microalga removed about 92% of 17α-ethinylestradiol (EE2) from the medium.

### 4.2. Bacterial Consortium

Increasing DEHP concentrations stimulated the growth of the bacterial consortium MP001. Accordingly, several studies have reported bacterial consortia’s high tolerance to DEHP because of their biodegradation ability [74,75,76,77,78,79,80,81,82,83,84]. Those microbial consortia were isolated from contaminated sediment and soil, sewage sludge, and wastewater [74,75,76,77,78,79,80,81,82,83,84], as well as MP001 consortium, which was obtained from mangrove sediment of Guanabara Bay (Rio de Janeiro, Brazil). Because of the high diversity of bacterial enzymes and metabolic pathways [85,86], strains and consortia are reported to break several xenobiotics down. Thus, bacteria use xenobiotics as a carbon source, which results in their biodegradation [87,88,89,90,91,92,93,94].

Regarding estimated growth rates of bacteria consortium, MP001 presented growth inhibition during all the exposure times at control incubation with mineral medium, indicating scarce resources. In the presence of DEHP, the growth rate of the consortium declined in the first 24 h and then increased after 48 h. This growth pattern observed in all concentrations suggests a selection for tolerant strains which may rise by degrading DEHP in place of sensitive strains. Once more, the consortium optical density declined after 72 h and enhanced after 96 h. We hypothesize that DEHP was degraded into intermediate compounds, which became a second selection factor of consortium strains. Hence, it seems that the consortium community changed through the exposure time according to the availability of compounds. Shifts in the bacterial community from other consortia after DEHP exposure have been observed [79,81,83]. Xenobiotics can modify the composition and structure of microbial communities, increasing the genera and/or families able to resist and use the compounds as a nutrient source [79,81,83]. Ningthoujam et al. [83] enriched a bacterial consortium able to degrade plasticizers from marine sediment. In this consortium, the major bacterial genera during degradation of the phthalate plasticizers were *Glutamicibacter*, *Ochrobactrum*, *Pseudomonas*, *Bacillus*, *Stenotrophomonas*, and *Methylophaga*. Whereas, in the presence of DEHP intermediates (mono-ethylhexyl phthalate—MEHP, 2-ethylhexanol, phthalic acid, and protocatechuic acid), the *Brevibacterium*, *Ochrobactrum*, *Achromobacter*, *Bacillus*, *Sporosarcina*, and *Microbacterium* populations enhanced [83].

Microbial consortia display more efficient degradation of organic pollutants than individual strains since they act through synergistic networks. Therefore, different strains degrade DEHP and its intermediate compounds by complementary pathways [79,81,83]. The degradation of DEHP includes phthalic acid, benzoic acid (or benzoate), catechol, and ring-cleavage pathways, depending on aerobic or anaerobic conditions [79,80,81,84]. Herein, bacteria optical density was significantly higher at mild DEHP concentrations (0.094, 0.187, 0.750, and 3.00 mg·L^−1^) compared with degradation studies where bacteria consortia were exposed to concentrations between 100 and 2000 mg·L^−1^ [78,82,83]. As the MP001 consortium showed a high tolerance to DEHP, its ability to degrade this phthalate should be investigated for further studies focusing on its application in bioremediation purposes. Additionally, the consortium strains should be identified to better understand community composition and function during the biodegradation process.

### 4.3. Zooplanktonic Grazer

Increases in DEHP concentrations raised *Artemia* sp. mortality, primarily at higher concentrations (1.50, 3.00, and 6.00 mg·L^−1^). However, this zooplanktonic crustacean showed some tolerance to DEHP since it did not reach 100% of mortality at any concentration within the tested exposure period. A mortality of 27% of individuals after 24 h of exposure to 6.00 mg DEHP·L^−1^ was registered herein. Although *Artemia* sp. is a model organism for ecotoxicological assays, studies testing phthalates toxicity on this crustacean are scarce. Almeida et al. [95] tested the toxicity of the plasticizer diethyl phthalate (DEP) against *Artemia salina* nauplii and found an LC_50,48h_ of 401.77 mg·L^−1^. DEP is a lower-molecular-weight plasticizer, and it is generally easier to degrade than higher-molecular-weight plasticizers like DEHP [4,5,96,97]. Therefore, it presents a low order of acute toxicity compared to DEHP, which explains the high LC_50_ concentration found for DEP [98,99]. In addition to DEHP synthetic production, some species of plants and bacteria assemble it as a secondary metabolite. *Artemia* sp. nauplii was exposed to DEHP coumpound produced by the flower *Calotropis gigantea*, and an LC_50,24h_ of 9.19 mg·L^−1^ was found [96]. This concentration estimated for nauplii stage is comparable to the findings of the present study. It is worth noting that the nauplii stages tend to be more sensitive than adults to contaminant exposure, including DEHP [95]. Henciya et al. [97] exposed *A. salina* nauplii and adults to a crude extract from a halophilic bacteria composed mostly of Bis(2-ethylhexyl) phthalate (27.7%), besides other chemical constituents. The authors found an LC_50,24h_ of 6 × 10^3^ mg·L^−1^ for nauplii and 2.4 × 10^4^ mg·L^−1^ for the adult stage. Moreover, after 24 h exposure, 100% of *A. salina* nauplii died at the concentration of 2.4 × 10^4^ mg·L^−1^ and adults showed 100% mortality at 1.92 × 10^5^ mg·L^−1^ of crude extract [100].

The sublethal effects evaluated in *Artemia* sp. seem applicable since this index increased according to concentration from 0.187 mg·L^−1^, which is close to environmentally relevant concentrations of DEHP (≤0.168 mg·L^−1^). Moreover, these indexes could be applied jointly or as an alternative endpoint for mortality assessment, mainly for soft responses at lower contaminant concentrations. Other ecotoxicological studies have already assessed alterations in the brine shrimp swimming speed [101]. Additionally, abnormal cells were growing on the necrotic part of the appendages. Indeed, DEHP was defined as potentially carcinogenic for humans [99]. A study carried out by Crobeddu et al. [102] demonstrated that DEHP could increase the proliferation of tumorous epithelial human cells at environmentally relevant concentrations and higher doses without cell deaths. These results corroborate the need to assess this sort of sublethal effect concomitantly to ecotoxicological assays.

### 4.4. Benthic Omnivorous Crustaceans

The survival of the amphipod *A. media* was significantly affected by the increasing concentrations of DEHP, and the exposure time intensified compound toxicity. After 24 and 48 h of exposure, amphipod mortality was higher in the lower DEHP concentrations (i.e., 0.045, 0.094, and 0.187 mg·L^−1^) compared with intermediate concentrations (i.e., 0.375, 0.750, and 1.50 mg·L^−1^). This response suggests a hormetic effect that corresponds to an overcompensation response to a disruption in homeostasis [100]. Hormesis is a widespread phenomenon in living beings (e.g., microbes, plants, invertebrates, and vertebrates) and occurs independently of the tested stressor and biological endpoint (reviewed in [103]). However, each biological system presents specific responses to a determined stressor (e.g., xenobiotic) or its different concentrations and time exposure. In previous ecotoxicological studies using amphipods, Green-Ojo et al. [104,105] did not report significant mortality of *Echingammarus marinus* (less than 20 and 17%, respectively) after 14 days of DEHP exposure (≤0.5 mg·L^−1^). DEHP concentration tested by previous studies corresponded to the intermediate concentrations in this study, which induced at least 40% of mortality (0.375 mg·L^−1^) after the first 24 h of exposure. Moreover, Yildirim et al. [106] found a LC_50,96h_ of 0.079 ± 0.01 mg DEHP·L^−1^ for the amphipod *Gammarus pulex*. The lethal concentration for *G. pulex* was within the range of concentrations tested here, and even the lowest DEHP concentration (0.045 mg·L^−1^) induced 66.67% of mortality after 24 h of exposure, reaching 100% after 96 h. After 72 and 96 h of exposure, no live amphipods were observed in almost all the DEHP concentrations. Therefore, *A. media* seems to be even more sensitive to DEHP than other amphipod species.

The absence of behavioral and morphological alterations during DEHP exposure may indicate strong molecular, tissue, and/or physiological effects that drove them to death before visible damages. Indeed, biochemical and molecular alterations often precede morphological damage after xenobiotic exposure, including phthalates [107,108,109]. In general, morphological endpoints take more time to be detected and are used during chronic exposure at low toxic concentrations. Yildirim et al. [106] showed oxidative stress, immunoreactivity, and tissue injury, primarily in the gills, of *G. pulex* individuals at extremely low DEHP concentrations (0.005, 0.01, and 0.02 mg·L^−1^) after 24 and 96 h of exposure. Regarding behavioral endpoints, Green-Ojo et al. [105] did not observe a significant effect on the swimming behavior and molting in *E. marinus* after 7 and 14 days (≤0.5 mg·L^−1^). However, the authors found a low observed effect concentration (LOEC) from 0.5 × 10^−3^ mg·L^−1^ for changes in precopulatory pairing behavior after 96 h of exposure [104]. M’Rabet et al. [74] emphasized the need for endpoints, including multiple biological levels for toxicity assessments—from molecular to physiological, morphological, and behavioral—to fully understand the impacts of contaminants on biota.

In addition to lethal and physiological responses, high concentrations of DEHP have been already registered in amphipods under natural conditions that were attributed to their feeding habits in combination with their low or lack of capacity for eliminating this compound [101,102]. Lo Brutto et al. [27] detected a mean of 0.046 ± 0.019 mg kg^−1^ of DEHP in five amphipod species from the Mediterranean Sea—*Talitrus saltator*, *Parhyale plumicornis*, *Parhyale aquilina*, *Speziorchestia stephenseni*, and *Orchestia montagui*. Martellini et al. [110] reported a similar mean concentration (0.035 ± 0.026 mg kg^−1^) in the species *T. saltator* from the Regional Natural Park of Migliarino (Pisa, Italy). Södergren [111] investigated DEHP accumulation and metabolism across different organisms (e.g., fish, invertebrates, and plants). The amphipod *Gammarus fossarum* (formerly known as *G. pulex*) accumulated the highest DEHP concentration among all tested organisms, reaching levels 24,500 times higher than those found in the water. Therefore, amphipod crustaceans seem to be useful as ecotoxicological models and bioindicators to detect and monitor contamination of plasticizers in coastal environments.

### 4.5. Suspensivorous Filter-Feeder

Herein, the first results of *Mytilopsis leucophaeata* exposure to DEHP are presented, since research on the effects of phthalates on *Mytilopsis* species is scarce. The studies previously conducted with bivalve species have shown DEHP tissue accumulation [35,112,113] and oxidative stress caused by the exposure to this organic pollutant, triggering antioxidant defense mechanisms [11,33,114,115]. Also, as a response to DEHP toxicity, peroxisomal and lysosomal alterations in digestive cells have been observed [114,116,117], besides immunological alterations in gene expression and hemocytes [35,118]. As an endocrine-disruptive chemical, DEHP has also affected reproduction events in *Mytilus edulis* [119,120].

In the ecotoxicological tests conducted here, the lethality of *M. leucophaeata* was observed from 72 h of exposure to DEHP at intermediate–high concentrations (i.e., 0.094, 0.187, 0.750, 1.50, and 3.00 mg·L^−1^), although no significant difference was noticed among treatments for this parameter. Most of the studies with bivalves exposure to DEHP have tested sublethal concentrations and reported no mortality during the assays, as follows: *Mytilus coruscus* exposed to 0.04–1.00 mg·L^−1^ for 28 days [112], *Mytilus galloprovincialis* at 0.5 mg·L^−1^ for 21 days [114], *Mytilus galloprovincialis* at 0.1 mg·L^−1^ for 21 days [117], *Mytilus edulis* exposed to 0.5 × 10^−3^ and 0.05 mg·L^−1^ for 7 days [119], and *Venerupis philippinarum* exposed to 0.4 and 4.0 mg·L^−1^ for 96 h [118]. The above-cited concentrations were close to or higher than those used in this study but mostly tested in longer assays. For this study, *M. leucophaeata* was sampled at Rodrigo de Freitas Lagoon, in which the presence of DEHP was reported in the sediment [19]. Additionally, microplastics have already been found in the dark false mussel tissues from the lagoon [41]. Hence, these individuals might have been previously exposed to low concentrations of DEHP and, consequently, this population may be less sensitive to this plasticizer, which may explain the lack of a significant effect on lethality. Nevertheless, *M. leucophaeata* was affected by this xenobiotic, and the lethality could be a workable endpoint to evaluate the short-term effects of DEHP depending on contaminant concentrations or previous exposure of the sampled population.

Regarding sublethal responses, dark false mussels exposed to higher DEHP concentrations (i.e., 0.750, 1.50, 3.00, and 6.00 mg·L^−1^) presented significantly lower sublethal indices with individuals completely closed or delayed in responding to the stimulus than those at control and lower toxicant concentrations (i.e., 0.045 and 0.187 mg·L^−1^). Similarly, Mincarelli et al. [119] did not observe significant differences in the opening and closing movements of *M. edulis* valves at 5 × 10^−4^ and 0.05 mg DEHP·L^−1^ when compared to the negative control. Behavioral responses are widely used as biomarkers of sublethal effects in bivalves for several classes of stressors [121,122,123]. Valve movements are used to monitor contaminants, as bivalves alter this behavior to counterbalance the stressor or isolate themselves from the water column. Therefore, prolonged valve closure usually indicates surrounding stressors [122]. Additionally, lower reaction capacity is related to the toxicity of some compounds to mussels and would make them more vulnerable in natural environments [121]. In this study, response to the stimulus was shown to be a good endpoint for ecotoxicological assays using *M. leucophaeata*, although it was significantly detected above environmentally relevant concentrations of DEHP (≤0.168 mg·L^−1^).

When exposed to higher DEHP concentrations (0.750, 1.50, 3.00, and 6.00 mg·L^−1^), dark false mussels significantly decreased byssus production compared with individuals from control and at lower concentrations (i.e., 0.045, 0.094, 0.187, 0.375 mg·L^−1^). However, after 72 h of incubation, byssus production by dark false mussels fell even at the control. Similarly, cluster behavior significantly fell at lower concentrations (i.e., 0.045, 0.094, and 0.187 mg·L^−1^) after 24 and 48 h of exposure. Then, after 72 h, cluster behavior fell in all treatments, including the control. Therefore, these sublethal endpoints were not appropriate to evaluate the DEHP effect after 72 h in *M. leucopheata*. This tendency to decrease byssus production over time might be related to previously reported metabolic alterations due to DEHP exposure [11,33,112,114,115,116,117,118,124], which may impair protein synthesis. Additionally, according to Rajagopal et al. [50], detached mussels showed less tolerance to xenobiotics since they presented higher oxygen consumption, filtration rate, foot activity, and byssus thread production [50]. These activities are associated with valve opening, making mussels more exposed to pollutants. Thus, a reduction in byssus production could corroborate with the mussel’s tendency in delaying the response to stimulus and lethality after 72 h of exposure to DEHP.

## 5. Conclusions

The five marine and brackish-water tropical species from distinct trophic levels were detected to have different sensitivities to acute exposure to DEHP. The microorganisms—a primary producer of microalga and a microbial consortium—showed high tolerance and a density-rising tendency during acute exposure to increasing DEHP concentrations, primarily at mild concentrations. These findings suggest that tested microorganisms might use this organic xenobiotic as a nutrient source. Therefore, these species might be considered as candidates for further studies focusing on removing and biodegradation analyses for bioremediation purposes in wastewater effluents, as well as in marine or brackish aquatic environments contaminated by DEHP. Among the invertebrates, the mortality of the filter-feeder zooplanktonic grazer *Artemia* sp. and the benthic omnivorous *A. media* rose with increasing DEHP concentrations. However, the amphipod *A. media* was more sensitive, primarily across the exposure time as the lethality reached 100% in almost all tested DEHP concentrations from 72 h. Hence, depending on the range of DEHP concentrations and exposure time, *Artemia* sp. was the more resistant crustacean species in ecotoxicological assays. Moreover, *Artemia* sp. presented sublethal responses to the tested DEHP concentrations, whereas *A. media* did not. The sublethal effects evaluated in *Artemia* sp. seem applicable since this index responded to concentrations from 0.187 mg·L^−1^, which are close to environmentally relevant concentrations of DEHP (≤0.168 mg·L^−1^). The filter-feeder bivalve *Mytilopsis leucophaeata* was the most tolerant invertebrate to DEHP. Dark false mussel lethality (≤20%) was observed exclusively after 72 h of exposure to DEHP at intermediate–high concentrations. However, its sublethal response to stimulus seems to be a good endpoint for ecotoxicological assays using the dark false mussels *M. leucophaeata*, although it was observed above environmentally relevant concentrations of DEHP. These results reinforce the evidence of DEHP contamination risks for tropical coastal ecosystems and, ultimately, for human beings. In addition to altering ecosystem dynamics by toxicity induced on biota, DEHP may accumulate throughout the food chain and poison the human beings since seafood consumption is the major route of human exposure to DEHP. DEHP is an endocrine-disruptive toxicant acting as an androgen antagonist in males; DHEP can also cause other adverse effects such as neurological disorders and cancer promotion. Hence, plastic and plasticizer contamination in environment, mainly in aquatic systems, must be avoided and controlled. Moreover, scientific findings should be considered by policymakers to lay down more effective regulations for the definition of safe environmental concentrations and potential risks of plasticizers for the biota, their sustainable use, and the discard of these compounds and related products.

## Figures and Tables

**Table 1:** Cumulative mortality (%) and standard deviation (±SD) of *Artemia* sp. individuals exposed to increasing DEHP concentrations.

DEHP (mg·L^−1^)	Cumulative Mortality (%)			
	24 h	48 h	72 h	96 h
0	3 (±6)	20 (±17)	27 (±21)	53 (±15)
0.045	10 (±10)	13 (±11)	13 (±11)	27 (±15)
0.094	10 (±10)	20 (±17)	30 (±10)	37 (±15)
0.187	10 (±17)	10 (±17)	23 (±15)	43 (±6)
0.375	13 (±6)	30 (±10)	33 (±23)	47 (±15)
0.750	13 (±23)	23 (±25)	47 (±6)	60 (±17)
1.50	20 (±17)	43 (±25)	63 (±25)	73 (±21)
3.00	23 (±15)	60 (±11)	47 (±6)	63 (±6)
6.00	27 (±21)	55 (±10)	70 (±10)	70 (±10)

**Table 2:** Index of sublethal effects detected in individuals of *Artemia* sp. exposed to increasing DEHP concentrations and their respective standard deviation (±SD). Data are presented as the mean value of replicates (n = 3 per treatment per time), where zero indicates the absence of sublethal effects among individuals exposed to concentration.

DEHP (mg·L^−1^)	Sublethal Index			
	24 h	48 h	72 h	96 h
0	0.00 (±0.00)	0.00 (±0.00)	0.00 (±0.00)	0.00 (±0.00)
0.045	0.00 (±0.00)	0.00 (±0.00)	0.00 (±0.00)	0.00 (±0.00)
0.094	0.07 (±0.13)	0.10 (±0.17)	0.00 (±0.00)	0.00 (±0.00)
0.187	0.00 (±0.00)	0.10 (±0.17)	0.15 (±0.26)	1.66 (±0.40)
0.375	0.00 (±0.00)	1.23 (±0.21)	0.58 (±0.23)	0.88 (±0.51)
0.750	0.03 (±0.06)	0.68 (±0.53)	0.82 (±0.17)	0.26 (±0.70)
1.50	0.19 (±0.33)	0.88 (±0.98)	0.22 (±0.38)	1.20 (±0.46)
3.00	0.35 (±0.30)	0.84 (±1.00)	0.53 (±0.92)	0.42 (±1.53)
6.00	0.40 (±0.70)	0.42 (±0.35)	1.53 (±1.52)	0.22 (±1.52)

**Table 3:** Cumulative mortality (%) and standard deviation (±SD) of *Apohyale media* individuals exposed to increasing DEHP concentrations.

DEHP (mg·L^−1^)	Cumulative Mortality (%)			
	24 h	48 h	72 h	96 h
0	0 (±0)	20 (±0)	53 (±42)	80 (±35)
0.045	67 (±23)	80 (±43)	93 (±12)	100 (±0)
0.094	60 (±53)	93 (±14)	100 (±0)	100 (±0)
0.187	73 (±12)	100 (±0)	100 (±0)	100 (±0)
0.375	40 (±0)	73 (±38)	100 (±0)	100 (±0)
0.750	20 (±20)	47 (±52)	73 (±31)	80 (±35)
1.50	47 (±12)	93 (±14)	100 (±0)	100 (±0)
3.00	100 (±0)	100 (±0)	100 (±0)	100 (±0)
6.00	100 (±0)	100 (±0)	100 (±0)	100 (±0)

**Table 4:** Cumulative mortality (%) and standard deviation (±SD) of *Mytilopsis leucophaeata* individuals exposed to increasing DEHP concentrations.

DEHP (mg·L^−1^)	Cumulative Mortality (%)			
	24 h	48 h	72 h	96 h
0	0 (±0)	0 (±0)	0 (±0)	0 (±0)
0.045	0 (±0)	0 (±0)	0 (±0)	0 (±0)
0.094	0 (±0)	0 (±0)	7 (±6)	7 (±6)
0.187	0 (±0)	0 (±0)	0 (±0)	7 (±6)
0.375	0 (±0)	0 (±0)	0 (±0)	0 (±0)
0.750	0 (±0)	0 (±0)	7 (±6)	7 (±6)
1.50	0 (±0)	0 (±0)	20 (±17)	20 (±17)
3.00	0 (±0)	0 (±0)	0 (±0)	7 (±6)
6.00	0 (±0)	0 (±0)	0 (±0)	0 (±0)

**Table 5:** Indices of sublethal effects in individuals of *Mytilopsis leucophaeata* exposed to different DEHP concentrations. Data are presented as the mean value and standard deviation (±SD) of independent replicates (n = 3 per treatment and time), where lower values indicate deleterious sublethal effects among individuals exposed to the concentration for the three indices.

DEHP (mg·L^−1^)	Response to Stimulus				Byssus Production				Cluster Formation			
	24 h	48 h	72 h	96 h	24 h	48 h	72 h	96 h	24 h	48 h	72 h	96 h
0	1.20 (±0.40)	0.73 (±0.30)	1.00 (±0.35)	0.67 (±0.12)	0.80 (±0.00)	0.80 (±0.00)	0.20 (±0.00)	0.00 (±0.00)	2.67 (±1.15)	2.67 (±1.15)	0.00 (±0.00)	0.00 (±0.00)
0.045	1.33 (±0.50)	1.00 (±0.40)	1.20 (±0.35)	0.53 (±0.42)	0.33 (±0.23)	0.20 (±0.20)	0.07 (±0.12)	0.13 (±0.23)	0.00 (±0.00)	0.00 (±0.00)	0.00 (±0.00)	0.67 (±1.15)
0.094	1.53 (±0.12)	1.17 (±0.60)	0.75 (±0.59)	0.55 (±0.40)	0.33 (±0.31)	0.60 (±0.35)	0.07 (±0.12)	0.00 (±0.00)	0.67 (±1.15)	1.33 (±1.15)	0.00 (±0.00)	0.00 (±0.00)
0.187	1.80 (±0.35)	0.87 (±0.61)	0.60 (±0.72)	0.88 (±0.33)	0.47 (±0.23)	0.47 (±0.23)	0.13 (±0.12)	0.07 (±0.12)	0.67 (±1.15)	0.67 (±1.15)	0.00 (±0.00)	0.00 ±0.00)
0.375	1.13 (±0.42)	1.00 (±0.53)	0.40 (±0.20)	0.87 (±0.58)	0.53 (±0.23)	0.53 (±0.12)	0.00 (±0.00)	0.07 (±0.12)	2.00 (±2.00)	2.33 (±2.08)	0.00 (±0.00)	0.00 (±0.00)
0.750	1.60 (±0.40)	1.13 (±0.31)	0.63 (±0.51)	0.53 (±0.76)	0.87 (±0.23)	0.80 (±0.20)	0.33 (±0.31)	0.07 (±0.12)	2.67 (±1.15)	2.67 (±1.15)	0.00 (±0.00)	0.00 (±0.00)
1.50	1.00 (±0.35)	1.33 (±0.64)	0.47 (±0.42)	0.63 (±0.32)	0.33 (±0.31)	0.53 (±0.42)	0.13 (±0.23)	0.00 (±0.00)	0.67 (±1.15)	1.33 (±2.3)	0.00 (±0.00)	0.00 (±0.00)
3.00	1.27 (±0.70)	0.60 (±0.35)	0.47 (±0.64)	0.80 (±0.35)	0.47 (±0.23)	0.47 (±0.12)	0.00 (±0.00)	0.00 (±0.00)	0.67 (±1.15)	0.67 (±1.15)	0.00 (±0.00)	0.00 (±0.00)
6.00	0.93 (±0.23)	0.80 (±0.00)	0.93 (±0.81)	0.53 (±0.76)	0.67 (±0.23)	0.60 (±0.20)	0.00 (±0.00)	0.07 (±0.12)	1.67 (±1.52)	1.67 (±1.52)	0.00 (±0.00)	0.00 (±0.00)

## Data Availability

The data presented in this study are available on request from the corresponding author.

## Supplementary Materials

The following supporting information can be downloaded at: https://www.mdpi.com/article/10.3390/ijerph22030402/s1, Mendeley Data, V1, https://doi.org/10.17632/y4r64wv9ry.1, Tables S1–S13: Groups that were significantly different depending on the interaction between DEHP concentrations (treatment) and time (24, 48, 72, and 96 h), according to pair-wise *posteriori* comparison.
