# Supplementary material for: The Threat of Bis(2-Ethylhexyl) Phthalate in Coastal and Marine Environments: Ecotoxicological Assays Using Tropical Species from Different Trophic Levels
Source: Int J Environ Res Public Health. 2025 Mar 10;22(3):402. doi: 10.3390/ijerph22030402 (PMC11942571; doi:10.3390/ijerph22030402)
Supplement: Supplementary file 1 [file ijerph-22-00402-s001.zip › ijerph-3461344-supplementary.pdf]

Supplementary data

Groups that were significantly different depending on the interaction between DEHP concentrations (treatment) and time (24, 48, 72, and 96 h), according to pair-wise *posteriori comparison*.

Table S1. DEHP concentration according to each treatment.

| Treatment | DEHP concentration (mg $L^{-1}$) |
|---|---|
| Control | 0.0 |
| 1 | 0.045 |
| 2 | 0.094 |
| 3 | 0.187 |
| 4 | 0.375 |
| 5 | 0.750 |
| 6 | 1.50 |
| 7 | 3.00 |
| 8 | 6.00 |

Mortality of the bacterial consortium MP001 (PERMANOVA, $F_{24,107} = 4.0589$, p = 0.0001)

Table S2. Tests among levels of the factor treatment within: level 1 of the factor time.

| Groups | *p* (Permanova) | *p* (Monte Carlo) |
|---|---|---|
| Control,3 | 0.1042 | 0.0075 |
| Control,7 | 0.0965 | 0.0136 |
| 1,3 | 0.0988 | 0.0410 |
| 1,7 | 0.0979 | 0.0436 |
| 2,3 | 0.1013 | 0.0004 |
| 2,4 | 0.1054 | 0.0216 |
| 2,5 | 0.0973 | 0.0093 |
| 2,6 | 0.1043 | 0.0200 |
| 2,7 | 0.1004 | 0.0024 |
| 3,8 | 0.1037 | 0.0125 |
| 5,8 | 0.0986 | 0.0423 |
| 6,8 | 0.1014 | 0.0472 |
| 7,8 | 0.1046 | 0.0133 |

Table S3. Tests among levels of the factor treatment within: level 2 of the factor time.

| Groups | *p* (Permanova) | *p* (Monte Carlo) |
|---|---|---|
| Control,1 | 0.0959 | 0.0053 |

| | | |
|---|---|---|
| Control,8 | 0.1015 | 0.0270 |
| 1,2 | 0.1032 | 0.0290 |
| 1,3 | 0.1047 | 0.0131 |
| 1,4 | 0.0989 | 0.0368 |
| 1,5 | 0.1097 | 0.0324 |
| 1,6 | 0.1025 | 0.0486 |
| 1,7 | 0.0972 | 0.0207 |
| 1,8 | 0.0993 | 0.0029 |
| 2,8 | 0.0953 | 0.0313 |
| 4,8 | 0.0943 | 0.0284 |

Table S4. Tests among levels of the factor treatment within: level 3 of the factor time.

| Groups | *p* (Permanova) | *p* (Monte Carlo) |
|---|---|---|
| 1,2 | 0.0971 | 0.0249 |
| 2,3 | 0.1008 | 0.0207 |
| 2,4 | 0.1007 | 0.0076 |
| 2,6 | 0.0986 | 0.0136 |
| 2,7 | 0.0950 | 0.0272 |
| 3,4 | 0.0993 | 0.0437 |

Table S5. Tests among levels of the factor treatment within: level 4 of the factor time.

| Groups | *p* (Permanova) | *p* (Monte Carlo) |
|---|---|---|
| Control,1 | 0.1002 | 0.0244 |
| Control,2 | 0.1016 | 0.0010 |
| Control,3 | 0.1008 | 0.0024 |
| Control,4 | 0.1011 | 0.0060 |
| Control,6 | 0.1001 | 0.0239 |
| Control,7 | 0.1086 | 0.0132 |
| 1,2 | 0.1010 | 0.0040 |
| 1,3 | 0.0984 | 0.0062 |
| 1,4 | 0.0964 | 0.0229 |
| 1,6 | 0.1029 | 0.0071 |
| 1,7 | 0.1030 | 0.0307 |
| 2,5 | 0.0930 | 0.0186 |
| 2,6 | 0.0991 | 0.0007 |
| 3,4 | 0.4948 | 0.5000 |
| 3,5 | 0.1018 | 0.0194 |
| 3,6 | 0.1012 | 0.0008 |
| 4,6 | 0.1015 | 0.0040 |
| 5,6 | 0.0982 | 0.0191 |
| 6,7 | 0.0991 | 0.0059 |

Mortality of the amphipod *Apohyale media* (PERMANOVA, $F_{24,107} = 4.1953$, p = 0.0001)

Table S6. Tests among levels of the factor treatment within: level 1 of the factor time.

| Groups | *p* (Permanova) | *p* (Monte Carlo) |
|---|---|---|
| Control,1 | 0.0001 | 0.0001 |
| Control,2 | 0.0001 | 0.0001 |
| Control,3 | 0.0001 | 0.0001 |
| Control,4 | 0.0001 | 0.0001 |
| Control,5 | 0.0001 | 0.0001 |
| Control,6 | 0.0001 | 0.0001 |
| Control,7 | 0.0001 | 0.0001 |
| Control,8 | 0.0970 | 0.0150 |
| 1,2 | 0.0001 | 0.0001 |
| 1,3 | 0.0001 | 0.0001 |
| 1,4 | 0.0001 | 0.0001 |
| 1,5 | 0.0001 | 0.0001 |
| 1,6 | 0.0001 | 0.0001 |
| 1,7 | 0.0001 | 0.0001 |
| 2,3 | 0.0001 | 0.0001 |
| 2,4 | 0.0001 | 0.0001 |
| 2,5 | 0.0001 | 0.0001 |
| 2,6 | 0.0001 | 0.0001 |
| 2,7 | 0.0001 | 0.0001 |
| 3,4 | 0.0001 | 0.0001 |
| 3,5 | 0.0001 | 0.0001 |
| 3,6 | 0.0001 | 0.0001 |
| 3,7 | 0.0001 | 0.0001 |
| 4,5 | 0.0001 | 0.0001 |
| 4,6 | 0.0001 | 0.0001 |
| 4,7 | 0.0001 | 0.0001 |
| 5,6 | 0.0001 | 0.0001 |
| 5,7 | 0.0001 | 0.0001 |
| 6,7 | 0.0001 | 0.0001 |

Table S7. Tests among levels of the factor treatment within: level 2 of the factor time.

| Groups | *p* (Permanova) | *p* (Monte Carlo) |
|---|---|---|
| Control,2 | 0.1018 | 0.0214 |
| Control,4 | 0.1027 | 0.0137 |
| Control,5 | 0.1015 | 0.0122 |
| Control,6 | 0.0993 | 0.0134 |

| | | |
|---|---|---|
| Control,7 | 0.1026 | 0.0126 |
| Control,8 | 0.1015 | 0.0102 |
| 1,4 | 0.0989 | 0.0035 |
| 1,5 | 0.1097 | 0.0043 |
| 1,6 | 0.1025 | 0.0040 |
| 1,7 | 0.0972 | 0.0053 |
| 1,8 | 0.0993 | 0.0040 |
| 2,4 | 0.0001 | 0.0001 |
| 2,5 | 0.0001 | 0.0001 |
| 2,6 | 0.0001 | 0.0001 |
| 2,7 | 0.0001 | 0.0001 |
| 2,8 | 0.0001 | 0.0001 |
| 4,5 | 0.0001 | 0.0001 |
| 4,6 | 0.0001 | 0.0001 |
| 4,7 | 0.0001 | 0.0001 |
| 4,8 | 0.0001 | 0.0001 |
| 5,6 | 0.0001 | 0.0001 |
| 5,7 | 0.0001 | 0.0001 |
| 5,8 | 0.0001 | 0.0001 |
| 6,7 | 0.0001 | 0.0001 |
| 6,8 | 0.0001 | 0.0001 |
| 7,8 | 0.0001 | 0.0001 |

Table S8. Tests among levels of the factor treatment within: level 3 of the factor time.

| Groups | $p$ (Permanova) | $p$ (Monte Carlo) |
|---|---|---|
| 1,5 | 0.1016 | 0.0004 |
| 1,6 | 0.1013 | 0.0004 |
| 1,7 | 0.1030 | 0.0004 |
| 1,8 | 0.0950 | 0.0006 |
| 5,6 | 0.0001 | 0.0001 |
| 5,7 | 0.0001 | 0.0001 |
| 5,8 | 0.0001 | 0.0001 |
| 6,7 | 0.0001 | 0.0001 |
| 6,8 | 0.0001 | 0.0001 |
| 7,8 | 0.0001 | 0.0001 |

Table S9. Tests among levels of the factor treatment within: level 4 of the factor time.

| Groups | $p$ (Permanova) | $p$ (Monte Carlo) |
|---|---|---|
| Control,1 | 0.1002 | 0.0021 |
| Control,7 | 0.1086 | 0.0016 |
| Control,8 | 0.0980 | 0.0023 |
| 1,7 | 0.0001 | 0.0001 |
| 1,8 | 0.0001 | 0.0001 |

| | | |
|---|---|---|
| 7,8 | 0.0001 | 0.0001 |

Byssus production of the mussel *Mytilopsis leucophaeata* (PERMANOVA, $F_{24,107}$ = 2.3960, p = 0.0023)

Table S10. Tests among levels of the factor treatment within: level 1 of the factor time.

| Groups | *p* (Permanova) | *p* (Monte Carlo) |
|---|---|---|
| Control,4 | 0.1015 | 0.0084 |
| Control,5 | 0.0984 | 0.0004 |
| Control,6 | 0.0973 | 0.0082 |
| Control,7 | 0.1000 | 0.0085 |
| Control,8 | 0.0986 | 0.0007 |
| 1,5 | 0.0988 | 0.0311 |
| 1,8 | 0.1066 | 0.0340 |
| 2,5 | 0.0980 | 0.0172 |
| 2,6 | 0.1983 | 0.0463 |
| 2,7 | 0.2052 | 0.0440 |
| 2,8 | 0.0978 | 0.0155 |
| 4,5 | 0.0993 | 0.0139 |
| 4,8 | 0.0990 | 0.0126 |

Table S11. Tests among levels of the factor treatment within: level 2 of the factor time.

| Groups | *p* (Permanova) | *p* (Monte Carlo) |
|---|---|---|
| Control,1 | 0.1984 | 0.0443 |
| Control,2 | 0.1019 | 0.0257 |
| 1,5 | 0.0994 | 0.0071 |
| 1,6 | 0.1010 | 0.0025 |
| 1,7 | 0.0983 | 0.0028 |
| 1,8 | 0.0998 | 0.0027 |
| 2,3 | 0.1002 | 0.0176 |
| 2,5 | 0.1001 | 0.0007 |
| 2,6 | 0.0001 | 0.0001 |
| 2,7 | 0.0001 | 0.0001 |
| 2,8 | 0.0001 | 0.0001 |
| 3,5 | 0.1014 | 0.0140 |
| 3,6 | 0.1038 | 0.0015 |
| 3,7 | 0.1018 | 0.0019 |
| 3,8 | 0.1035 | 0.0009 |
| 4,5 | 0.0993 | 0.0236 |
| 4,6 | 0.1004 | 0.0058 |

| | | |
|---|---|---|
| 4,7 | 0.0934 | 0.0073 |
| 4,8 | 0.1061 | 0.0064 |
| 6,7 | 0.0001 | 0.0001 |
| 6,8 | 0.0001 | 0.0001 |
| 7,8 | 0.0001 | 0.0001 |

Table S12. Tests among levels of the factor treatment within: level 3 of the factor time.

| Groups | *p* (Permanova) | *p* (Monte Carlo) |
|---|---|---|
| 2,3 | 0.0980 | 0.0224 |
| 3,4 | 0.0999 | 0.0056 |
| 3,5 | 0.0975 | 0.0022 |
| 3,6 | 0.1057 | 0.0024 |
| 3,7 | 0.1093 | 0.0050 |
| 3,8 | 0.1055 | 0.0021 |
| 4,5 | 0.0001 | 0.0001 |
| 4,6 | 0.0001 | 0.0001 |
| 4,8 | 0.0001 | 0.0001 |
| 5,6 | 0.0001 | 0.0001 |
| 5,8 | 0.0001 | 0.0001 |
| 6,8 | 0.0001 | 0.0001 |

Table S13. Tests among levels of the factor treatment within: level 4 of the factor time.

| Groups | *p* (Permanova) | *p* (Monte Carlo) |
|---|---|---|
| Control, 6 | 0.0987 | 0.0233 |
| 1,6 | 0.1004 | 0.0248 |
| 2,6 | 0.1019 | 0.0432 |
